# Human Cardiac Function Simulator for the Optimal Design of a Novel Annuloplasty Ring with a Sub-valvular Element for Correction of Ischemic Mitral Regurgitation

**DOI:** 10.1007/s13239-015-0216-z

**Published:** 2015-02-07

**Authors:** Brian Baillargeon, Ivan Costa, Joseph R. Leach, Lik Chuan Lee, Martin Genet, Arnaud Toutain, Jonathan F. Wenk, Manuel K. Rausch, Nuno Rebelo, Gabriel Acevedo-Bolton, Ellen Kuhl, Jose L. Navia, Julius M. Guccione

**Affiliations:** 1 Dassault Systèmes Simulia Corporation, Fremont, CA USA; 2 Graduate Program in Material Science (PPG-CIMA), Faculty UnB Planaltina, University of Brasilia, Brasília, Brazil; 3 Department of Radiology and Biomedical Imaging, University of California at San Francisco, San Francisco, USA; 4 Department of Mechanical Engineering, Michigan State University, East Lansing, MI USA; 5 Institute for Biomedical Engineering, University and ETH Zürich, Zurich, Switzerland; 6 Department of Surgery, University of California at San Francisco, San Francisco, USA; 7 Department of Mechanical Engineering, University of Kentucky, Lexington, KY USA; 8 Departments of Mechanical Engineering, Bioengineering and Cardiothoracic Surgery, Stanford University, Stanford, CA USA; 9 Department of Cardiovascular and Thoracic Surgery, Cleveland Clinic, Cleveland, OH USA; 10 Division of Adult Cardiothoracic Surgery, Department of Surgery, School of Medicine, Mount Zion Harold Brunn Institute for Cardiovascular Research, UCSF, 1657 Scott St., Room 219, San Francisco, CA 94143 USA

**Keywords:** Finite element method, Realistic simulation, Myocardial infarction, Ventricular function, Ischemic mitral regurgitation, Mitral annuloplasty

## Abstract

Ischemic mitral regurgitation is associated with substantial risk of death. We sought to: (1) detail significant recent improvements to the Dassault Systèmes human cardiac function simulator (HCFS); (2) use the HCFS to simulate normal cardiac function as well as pathologic function in the setting of posterior left ventricular (LV) papillary muscle infarction; and (3) debut our novel device for correction of ischemic mitral regurgitation. We synthesized two recent studies of human myocardial mechanics. The first study presented the robust and integrative finite element HCFS. Its primary limitation was its poor diastolic performance with an LV ejection fraction below 20% caused by overly stiff* ex vivo* porcine tissue parameters. The second study derived improved diastolic myocardial material parameters using *in vivo* MRI data from five normal human subjects. We combined these models to simulate ischemic mitral regurgitation by computationally infarcting an LV region including the posterior papillary muscle. Contact between our novel device and the mitral valve apparatus was simulated using Dassault Systèmes SIMULIA software. Incorporating improved cardiac geometry and diastolic myocardial material properties in the HCFS resulted in a realistic LV ejection fraction of 55%. Simulating infarction of posterior papillary muscle caused regurgitant mitral valve mechanics. Implementation of our novel device corrected valve dysfunction. Improvements in the current study to the HCFS permit increasingly accurate study of myocardial mechanics. The first application of this simulator to abnormal human cardiac function suggests that our novel annuloplasty ring with a sub-valvular element will correct ischemic mitral regurgitation.

**Electronic supplementary material:**

The online version of this article (doi:10.1007/s13239-015-0216-z) contains supplementary material, which is available to authorized users.

## Introduction

Ischemic mitral regurgitation is a consequence of adverse left ventricular (LV) remodeling after myocardial injury, in which there is enlargement of the LV chamber and mitral annulus, apical and lateral migration of the papillary muscles, leaflet tethering, and reduced coaptation area. These processes lead to malcoapation of the leaflets and consequently mitral insufficiency. The leaflets themselves are normal, and the disease occurs in the myocardium rather than in the valve itself.^2^ Despite this, beyond medical therapy, surgical correction of mitral regurgitation has largely relied upon native valve repair or valve replacement. A recent randomized clinical trial^2^ found no significant difference in LV reverse remodeling or survival at 12 months between patients who underwent mitral-valve repair with ring annuloplasty and those who underwent mitral-valve replacement. Replacement provided a more durable correction however, with mitral regurgitation recurring less than 1/10th as often at 12 months compared to outcomes of valve repair. We hypothesize that mitral-valve repair using a novel annuloplasty ring with a sub-valvular element would provide a more effective treatment of ischemic mitral regurgitation than mitral-valve repair using a standard annuloplasty ring. Our rationale is that the sub-valvular element engages the chordae of the posterior leaflet, and contacts the inferior free edge of said leaflet. As a consequence, it counterbalances an apical and lateral shifting of the coaptation line in patients with ischemic cardiomyopathy, thus reestablishing proper leaflet coaptation and valve function.

Computational modeling is one way to systematically assess the effects of pathology and proposed surgical repair on heart function.^11^ By simulating the effect of disease or injury on the heart, simulations allow investigators to explore a variety of pathologic conditions that would be difficult to consistently create *in vivo*.^6^ Models also have an important role in cardiac device design, as application of myriad treatment designs to identical pathology allows for natural isolation of treatment effects.^8^, ^18^ However, current models of cardiac function are limited by assumptions related to geometry and material properties, and importantly, none have been fully validated with detailed experimental data.^17^ Dassault Systèmes SIMULIA recently launched the Living Heart Project to develop an integrated physics-based simulator of cardiac physiology. The Living Heart model or Human Cardiac Function Simulator (HCFS) not only includes the entire heart—all four chambers, valves and major vessels—but also includes the electrophysiology and fibrous architecture of the myocardium. There is increasing evidence that this model can be used to predict the progression of pathology and the outcome both of surgical repair and use of cardiac devices. We recently published a proof-of-concept methods paper using this model to simulate the normal human heart.^3^ Although groundbreaking, the initial model had some limitations. Most importantly, the LV ejection fraction was only 19%, much lower than the normal range at rest (62.3 ± 6.1%) as assessed using radionuclide angiocardiography.^14^ In the current study, we simulated a physiologically normal LV ejection fraction by using more realistic heart geometry and diastolic myocardial material properties than those proposed in the earlier proof-of-concept paper.^3^

In this invited article concerned with mitral valve function, pathology and therapeutic options, we therefore seek to: (1) detail our significant improvements in the geometry and diastolic material properties of the Dassault Systèmes HCFS; (2) use the resulting more realistic model to simulate normal cardiac function as well as abnormal cardiac function due to a myocardial infarction that includes the posterior LV papillary muscle; and (3) use the abnormal human heart model to debut a novel annuloplasty ring with a sub-valvular element for correction of ischemic mitral regurgitation.

## Materials and Methods

### Human Cardiac Function Simulator

**Figure 1:**
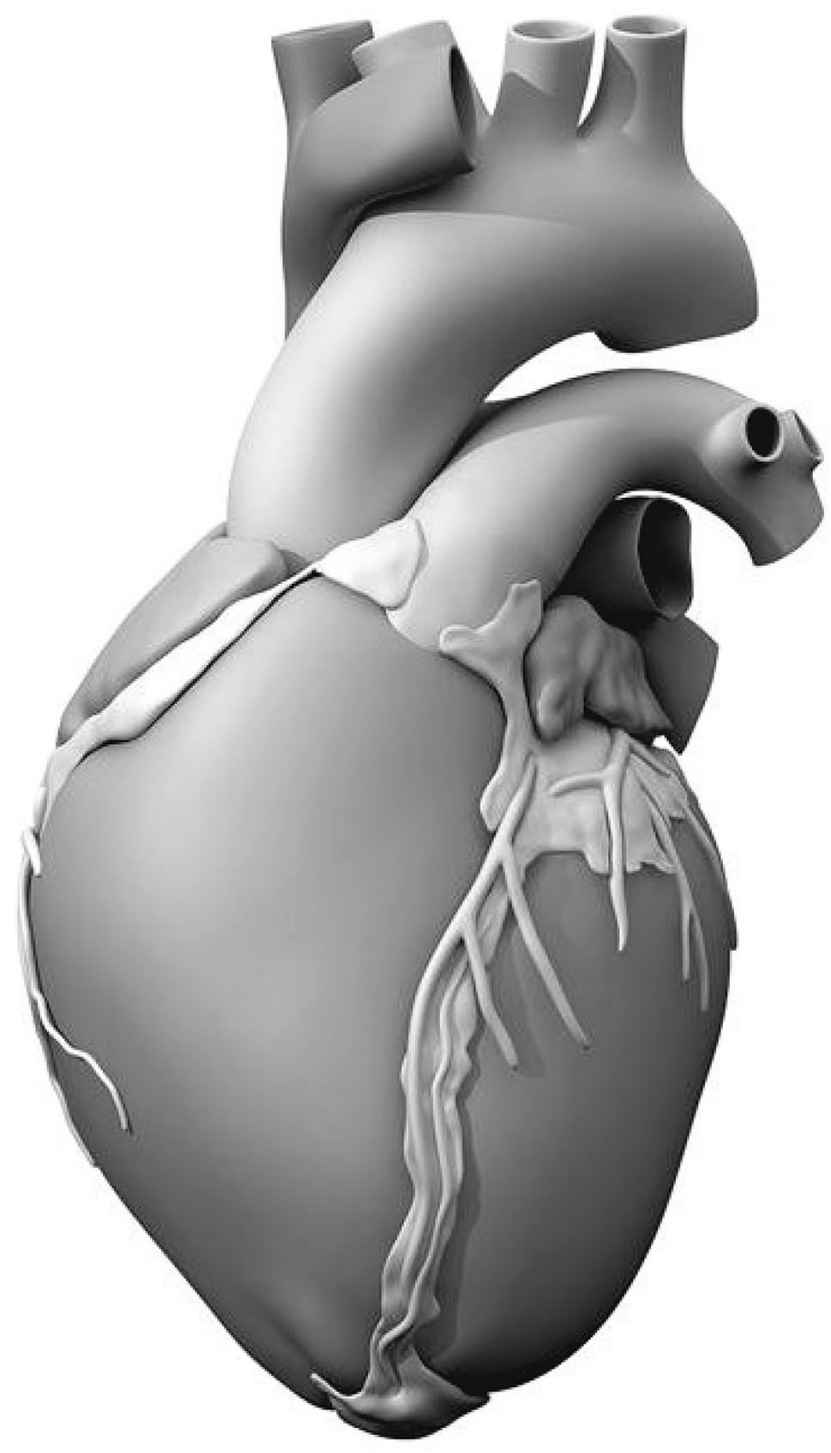
Solid model of the human heart used as the basis for our improved model. It was created from computed tomography and magnetic resonance imaging; adapted with permission from (Zygote Media Group and Inc., 2014)

We refer the interested reader to Sect. 2 of Baillargeon *et al*.^3^ for our continuum model of electro-mechanical coupling based on the kinematic equations, the balance equations, and the constitutive equations. In Sect. 3 of that methods paper, we illustrate our computational model, based on the strong and weak forms of the governing equations, their temporal and spatial discretizations, their linearizations, and the handling of internal variables. In the present study, we use a more realistic solid model of the human heart (Fig. 1).

The Solid Zygote 3D Heart geometries for computer-aided design (CAD) and simulation were created by Zygote Media Group, Inc. (UT, USA). Generation I of the Solid Zygote Heart geometry (the one we used in Ref. 3) was developed using medical imaging data from a single human subject in 2005. An 8-slice GE Light Speed CT platform was used to acquire 5 mm axial slices, with an imaging matrix of 512 × 512. Generation II of the Solid Zygote Heart geometry (the one used in the current study) was created by the Zygote Media Group in 2013, using improved imaging techniques. A 64-slice GE Light Speed scanner, using retrospective ECG gating and achieving a slice thickness of 0.75 mm with 512 × 512 imaging matrix, was used to acquire images of an anonymous healthy middle-aged Caucasian male. Imaging data was resampled to twice the voxel density in each dimension, reformatted into standard cardiac imaging views, and was manually segmented to create a rough polygonal geometry. This rough geometry was then cleaned of noise, artifacts, and anomalies using standard 3D editing software (Symbolics/Nichiman Graphics/Mirai, Wavefront, 3D Studio/3D Studio Max, Alias Power Animator, Maya, Cinema 4D, Focus, Amira, and SolidWorks). Just as for the first generation Solid Zygote 3D Heart, certain anatomic elements (e.g., papillary muscles, valves, and chordae tendineae) had to be added to the model manually, as their fine detail could not be captured well with clinical imaging technology. In the same respect, the finest geometric details of certain anatomical structures (e.g., trabeculae carneae) were simplified so as to avoid an unnecessarily complex and computationally demanding model. It is important to note here that the Generation II Solid Zygote Heart geometry is a component of a larger anatomical collection that includes integrated anatomical geometries of a 50th percentile (U.S.) male and hence, there are no “patient-specific” anatomical features of the heart model.

**Figure 2:**
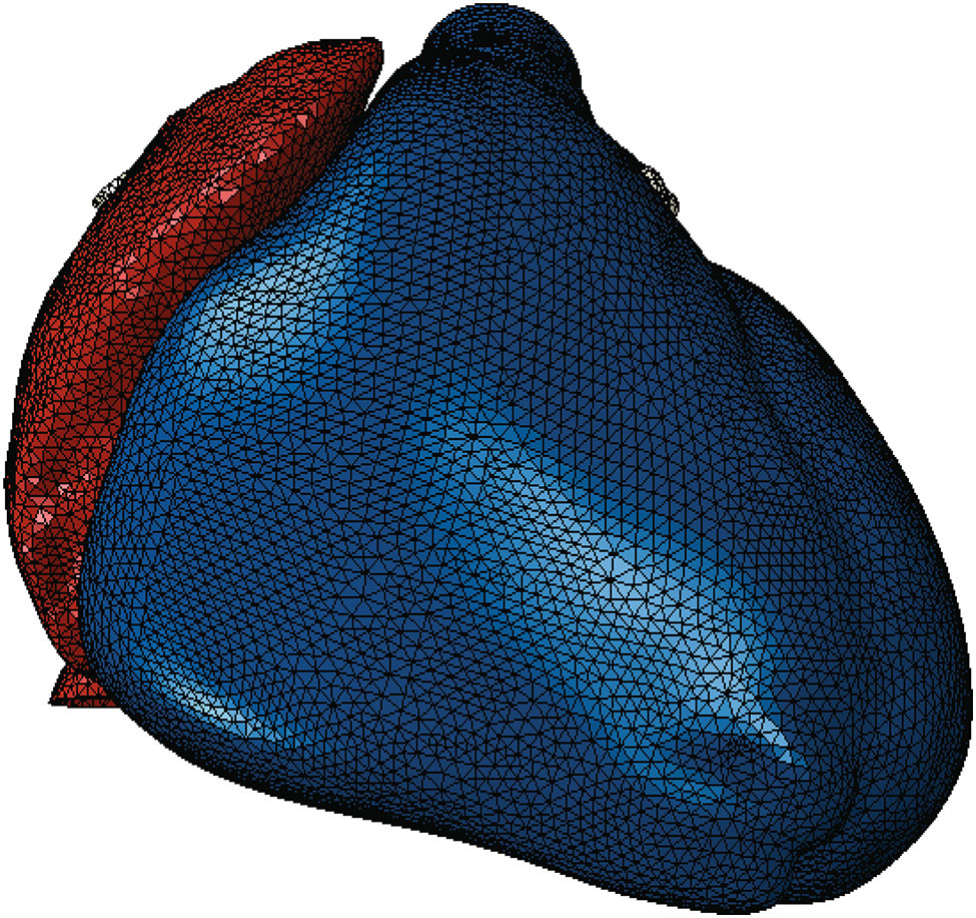
Electrical finite element model of the human heart discretized with 449,560 linear tetrahedral elements, 655 1D linear conduction elements, 103,770 nodes, and 103,770 electrical degrees of freedom. Red elements comprise the atria; blue elements comprise the ventricles

**Figure 3:**
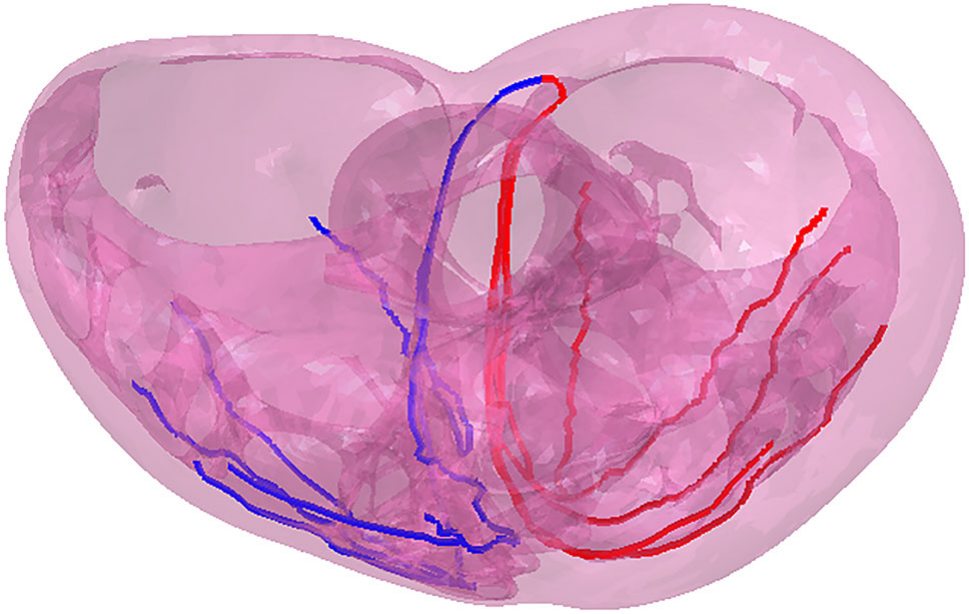
Representation of the 655 1D linear electrical conduction elements in the LV (red) and RV (blue) used in the HCFS

**Figure 4:**
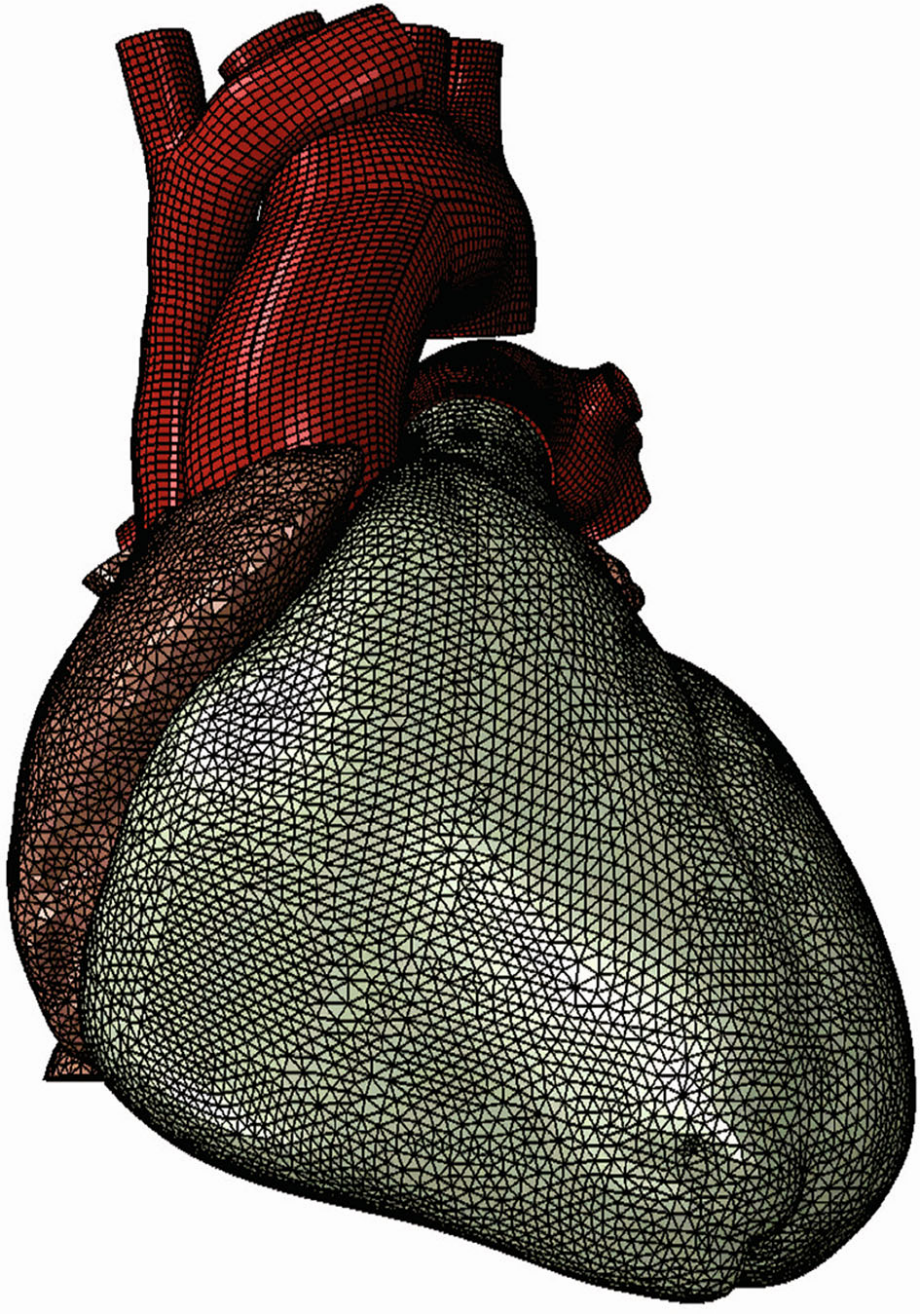
Mechanical finite element model of the human heart discretized with 449,560 linear tetrahedral elements, 12,915 linear quadrilateral shells, 7577 linear triangular shells, 636 linear truss elements, 16,824 rigid triangular elements, 130,290 nodes, and 443,564 mechanical degrees of freedom

Figure 2 shows the electrical finite element model of Zygote’s second-generation solid heart geometry discretized with 449,560 linear tetrahedral elements, 655 1D linear conduction elements, 103,770 nodes, and 103,770 electrical degrees of freedom. The model depicted in that figure was created using Abaqus CAE. Figure 3 shows how the 655 1D linear electrical conduction elements are distributed between the left and right ventricles. Figure 4 shows the mechanical finite element model of the human heart discretized with 449,560 linear tetrahedral (C3D4) elements, 12,915 linear quadrilateral shells, 7577 linear triangular (S4R reduced integration) shells, 636 linear truss (T3D2) elements, 16,824 rigid triangular elements, 130,290 nodes, and 443,564 mechanical degrees of freedom. The S4R is the only element type with hourglass control (based on enhanced strain). Otherwise, the muscle fiber model, the fluid model, and most of the model parameters in the current study are as described in Sect. 4 of Baillargeon *et al*.^3^

**Figure 5:**
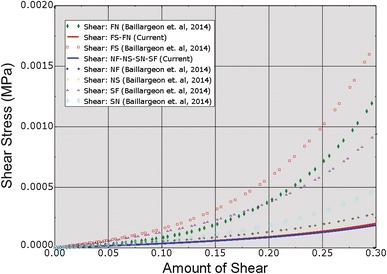
Comparison of passive mechanical behavior under shearing deformation between the human heart model of Baillargeon *et al*.^3^ and that used in the current study. “FN” stands for shear in the fiber-normal plane. “FS” stands for shear in the fiber-sheet plane. True (Cauchy) stress and true (logarithmic) strain are plotted in this figure

The only non-valvular model parameters used in the current study that differ from those in Table 1 of Baillargeon *et al*.^3^ relate to the passive material properties of myocardium, and are as follows: *k* = 1000 kPa; *a* = 0.33 kPa; *b* = 7.08; *a*_ff_ = 0.25 kPa; *b*_ff_ = 5.34; *a*_ss_ = 0; *b*_ss_ = 0; *a*_fs_ = 0; *b*_fs_ = 0. Our rationale for modifying these parameters is to correct the non-physiologic LV ejection fraction of the first generation HCFS. In Baillargeon *et al*.^3^ we used model parameter values from the literature (see references listed in their Table 1) concerning electrical, mechanical, and electro-mechanical phenomena and flow in the heart, and modified unknown model parameter values concerning circulation to simulate an LV pressure–volume loop. The resulting LV pressures and shape of the loop in Fig. 10 of that study are realistic, but the LV volumes are not indicative of a normal human heart. Specifically, end-diastolic and end-systolic volumes were 103 and 83 mL, respectively, corresponding to a stroke volume of 20 mL and an ejection fraction of only 19.4% (=20 mL divided by 103 mL). In a recent study by Genet *et al*.^8^ five normal human subject-specific LV models were created, with an average ejection fraction of 56 ± 3.95%. In the current study, the passive mechanical model parameter values were obtained by fitting the passive mechanical behavior under shearing deformation obtained from the constitutive model for the normal myocardium in Appendix A of Genet *et al*.^8^ Figure 5 shows a comparison of passive mechanical behavior under shearing deformation between the human heart model of Baillargeon *et al*.^3^ and the one used in the current study.

**Figure 6:**
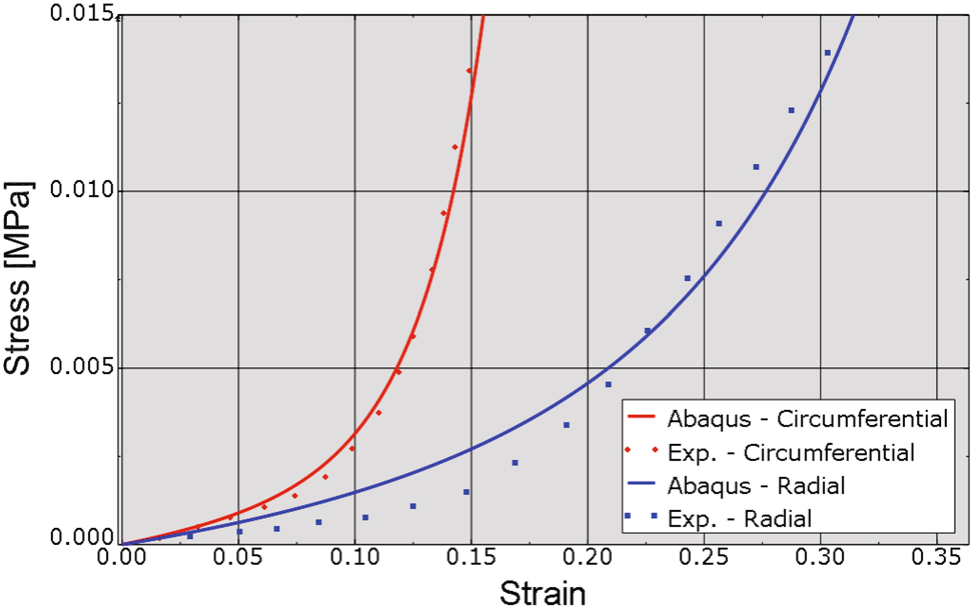
Fit of leaflet stress–strain relationships (red indicates circumferential, blue indicates radial) to experimental data. “Exp” stands for experimental. True (Cauchy) stress and true (logarithmic) strain are plotted in this figure

**Figure 7:**
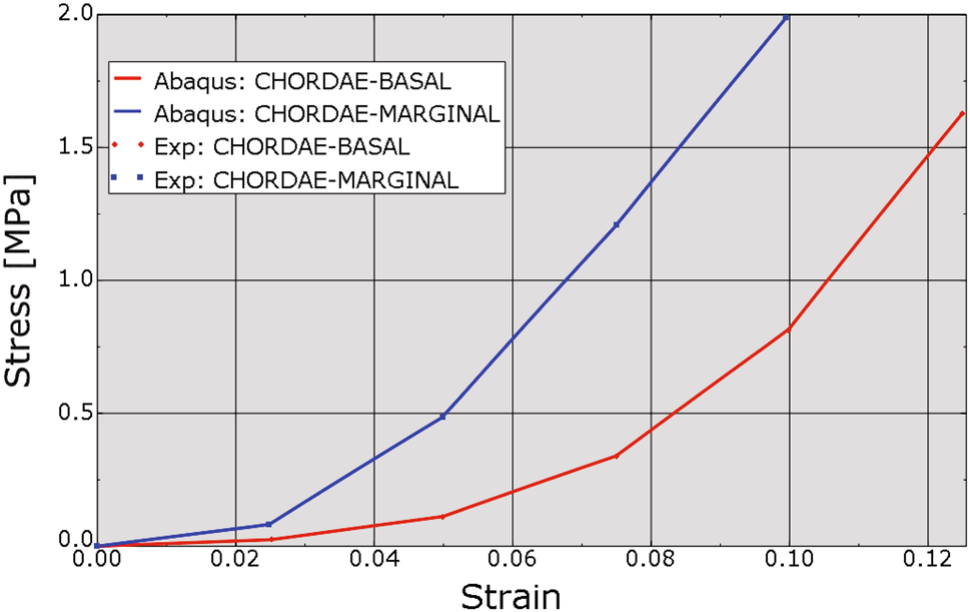
Fit of chordae stress–strain relationships to experimental data. Red line indicates basal chordae; blue line indicates marginal chordae. “Exp” stands for experimental. True (Cauchy) stress and true (logarithmic) strain are plotted in this figure

Since the current study was mainly concerned with mitral valve function, pathology and therapeutic options, additional attention was given to the mechanical properties of the leaflets and chordae. Figure 6 shows the fit of model parameter values defining leaflet circumferential and radial stress–strain relationships to measurements from the study of May-Newman and Yin.^13^ Thus, porcine data was used to simulate human mitral tissue. Similarly, Fig. 7 shows the fit of model parameter values defining chordae basal and marginal stress–strain relationships to measurements from the study of Kunzelman and Cochran.^10^ In both cases, the Dassault Systèmes SIMULIA optimization software Isight was used to minimize least-squared differences between measured and predicted stresses at matching strains.

### Human Cardiac Function Simulator with LV Myocardial Infarction

**Figure 8:**
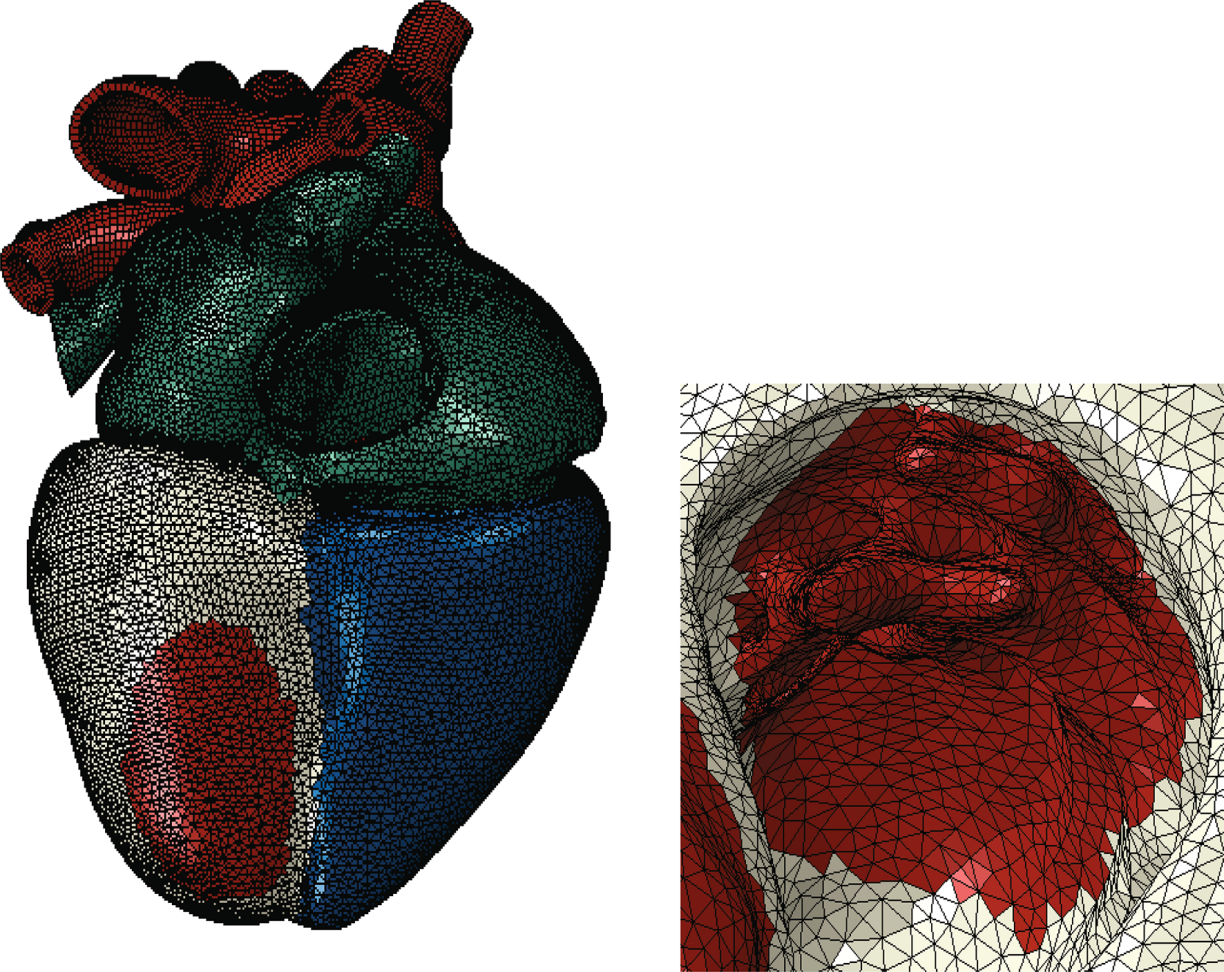
Ischemic mitral regurgitation was simulated by preventing active myocardial stress to be developed in an LV region (shown in red). Left: view of whole heart model; Right: close-up view of infarcted LV region showing endocardial surface and posterior papillary muscle

The state-of-the-art whole human heart model described above was then modified in the most straightforward manner to simulate regurgitant mitral valve mechanics. This modification involved creating an LV region that included the posterior papillary muscle. The myocardial material properties in that region were modified to ensure that no active myocardial stress was generated at any time during the cardiac cycle, thus simulating infarction. Figure 8 shows the LV region in which active myocardial stress development was suppressed.

### Novel Device for Correction of Ischemic Mitral Regurgitation

**Figure 9:**
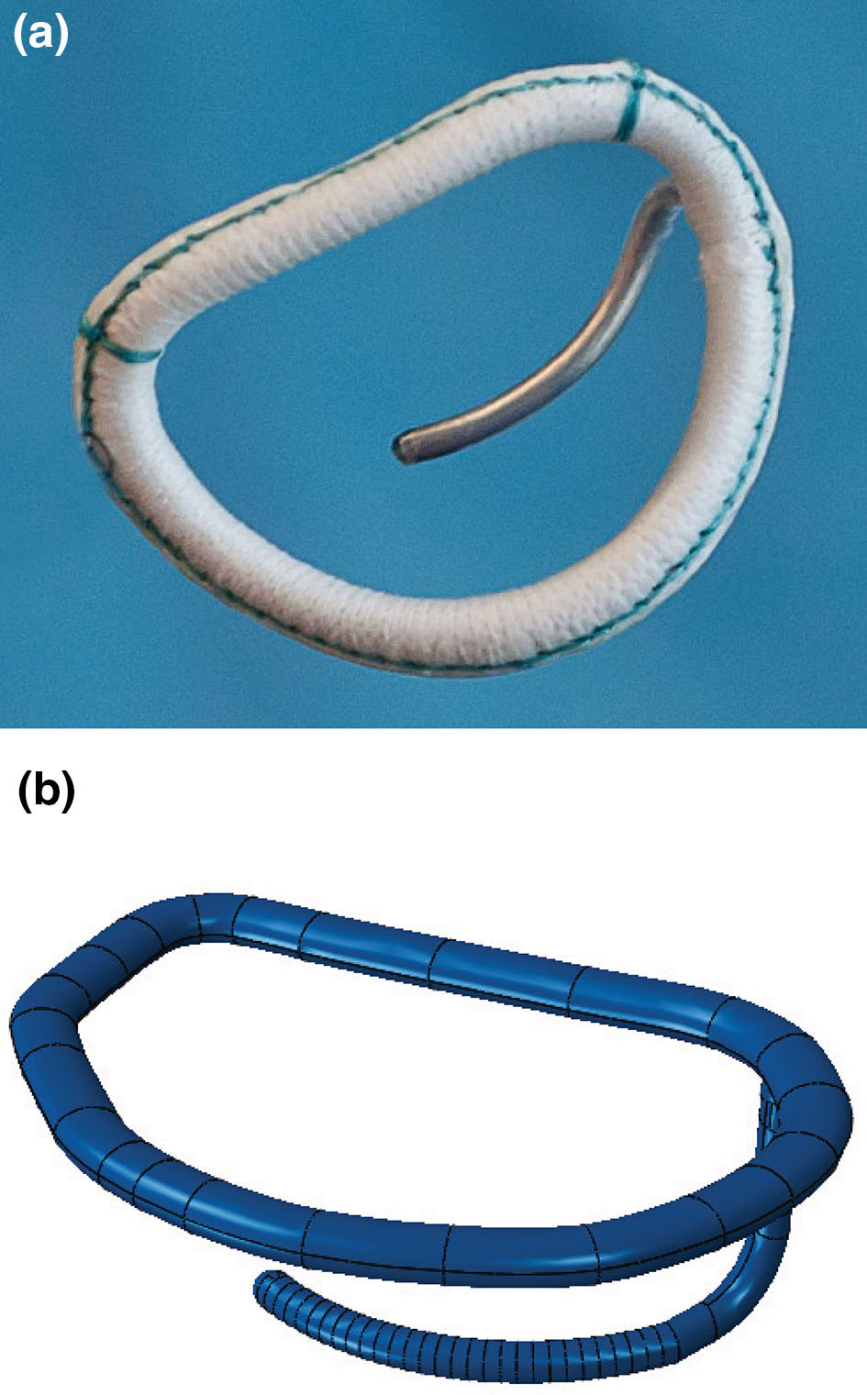
Novel annuloplasty ring with a sub-valvular element for correction of ischemic mitral regurgitation. a Photograph of actual prototype. b Finite element model of prototype

**Figure 10:**
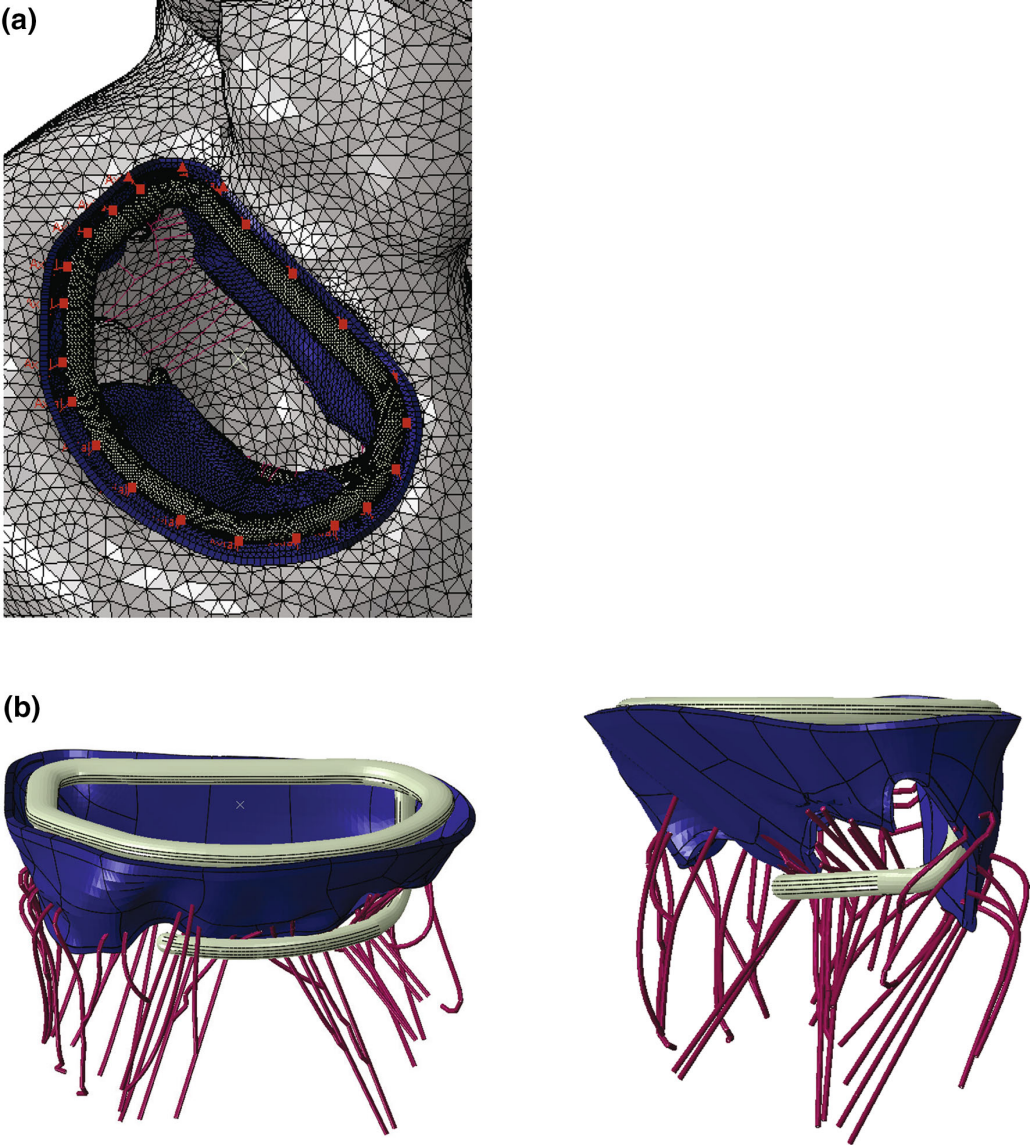
a Close-up view of whole heart model with left atrium removed. Sutures attaching the novel device to the mitral valve annulus are shown in pink. b Side views showing finite element model of novel device in contact with mitral valve apparatus

A recent randomized clinical trial^2^ confirmed an excess of incidence of recurrence of mitral regurgitation at 1 year among patients undergoing mitral-valve repair. The type of annuloplasty ring was based on the surgeon’s preference. The protocol mandated the use of an approved rigid or semi-rigid complete annuloplasty ring, which was downsized for the annulus diameter. In collaboration with the Cleveland Clinic (Ohio, USA) a prototype annuloplasty ring with a sub-valvular element was developed (Fig. 9a). To the best of our knowledge, it is the first annuloplasty ring to include a sub-valvular element. Our rationale is that the sub-valvular element engages the chordae of the posterior leaflet, and contacts the inferior free edge of said leaflet. Consequently, it counterbalances an apical and lateral shifting of the coaptation line in patients with ischemic cardiomyopathy, thus reestablishing proper leaflet coaptation and valve function. We created a rigid finite element representation of this novel device, which is shown in Fig. 9b. The device was modeled as rigid for simplicity. Figure 10a is a close-up view of the whole heart model with the left atrium removed. Figure 10b clearly illustrates the computational geometry of our novel device in contact with the mitral valve apparatus. Tight, frictionless contact between the device and the mitral valve annulus was activated at numerous points that represented sutures. It is important to point out here that there was separation contact (separation was allowed); a standard penalty-based contact algorithm was used.

## Results

**Figure 11:**
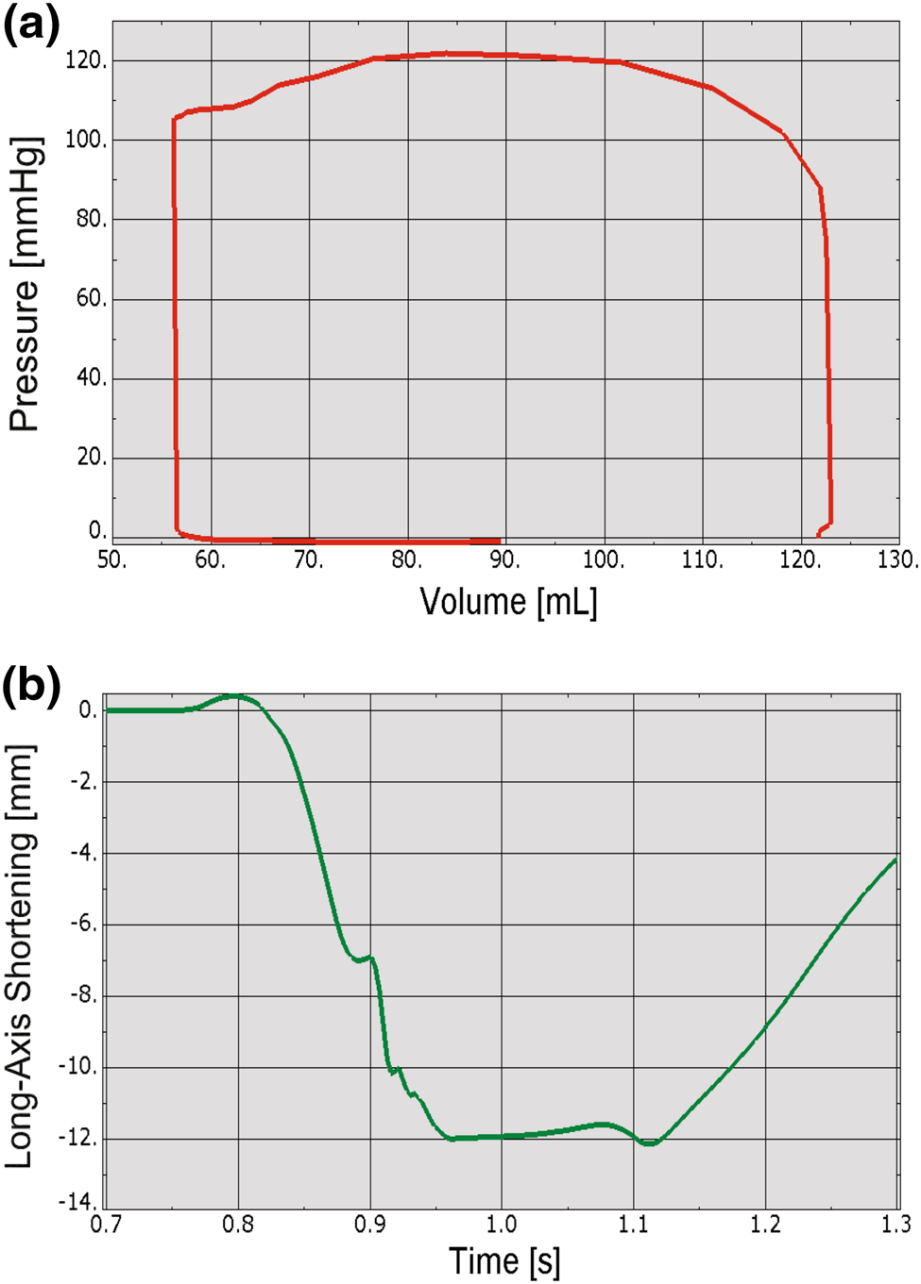
Plots showing normal human heart behavior. a LV pressure–volume loop. b LV long-axis shortening vs. time

**Figure 12:**
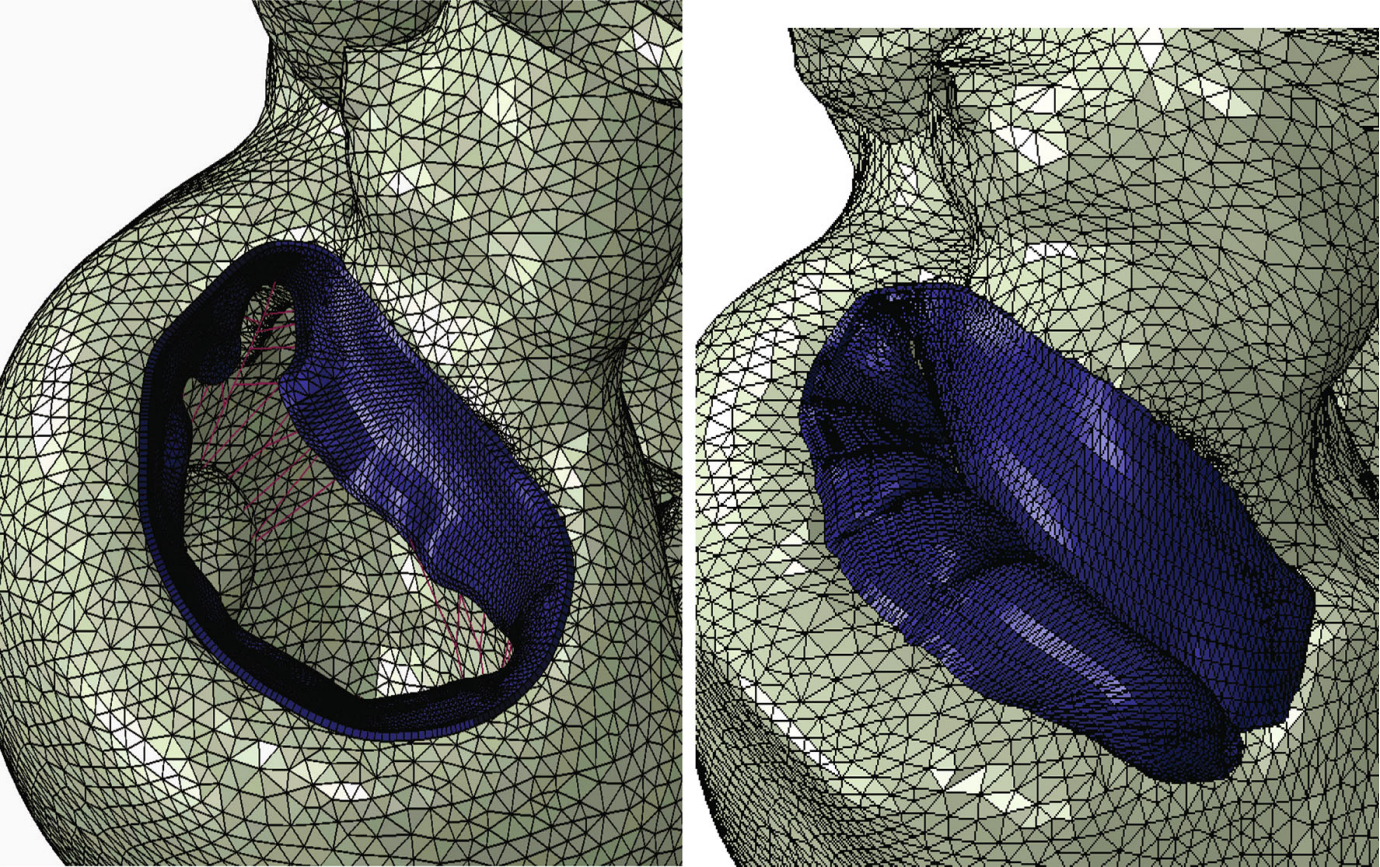
Close-up views of whole heart model with left atrium removed to clearly show mitral valve in its open (left) and closed (right) states during the cardiac cycle

**Figure 13:**
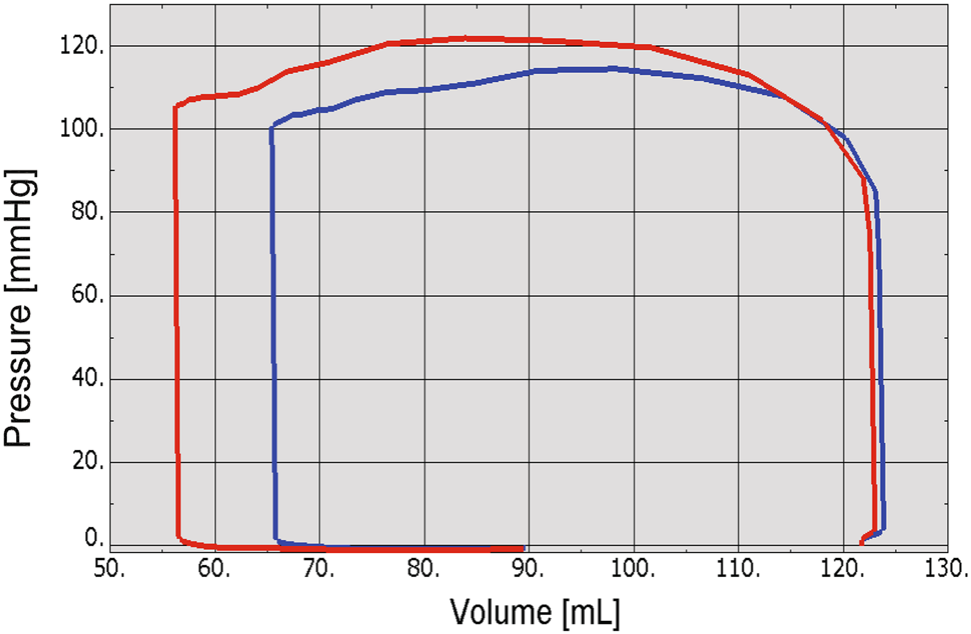
Effect of myocardial infarction (blue line) on LV pressure–volume loop. The red line indicates no myocardial infarction

**Figure 14:**
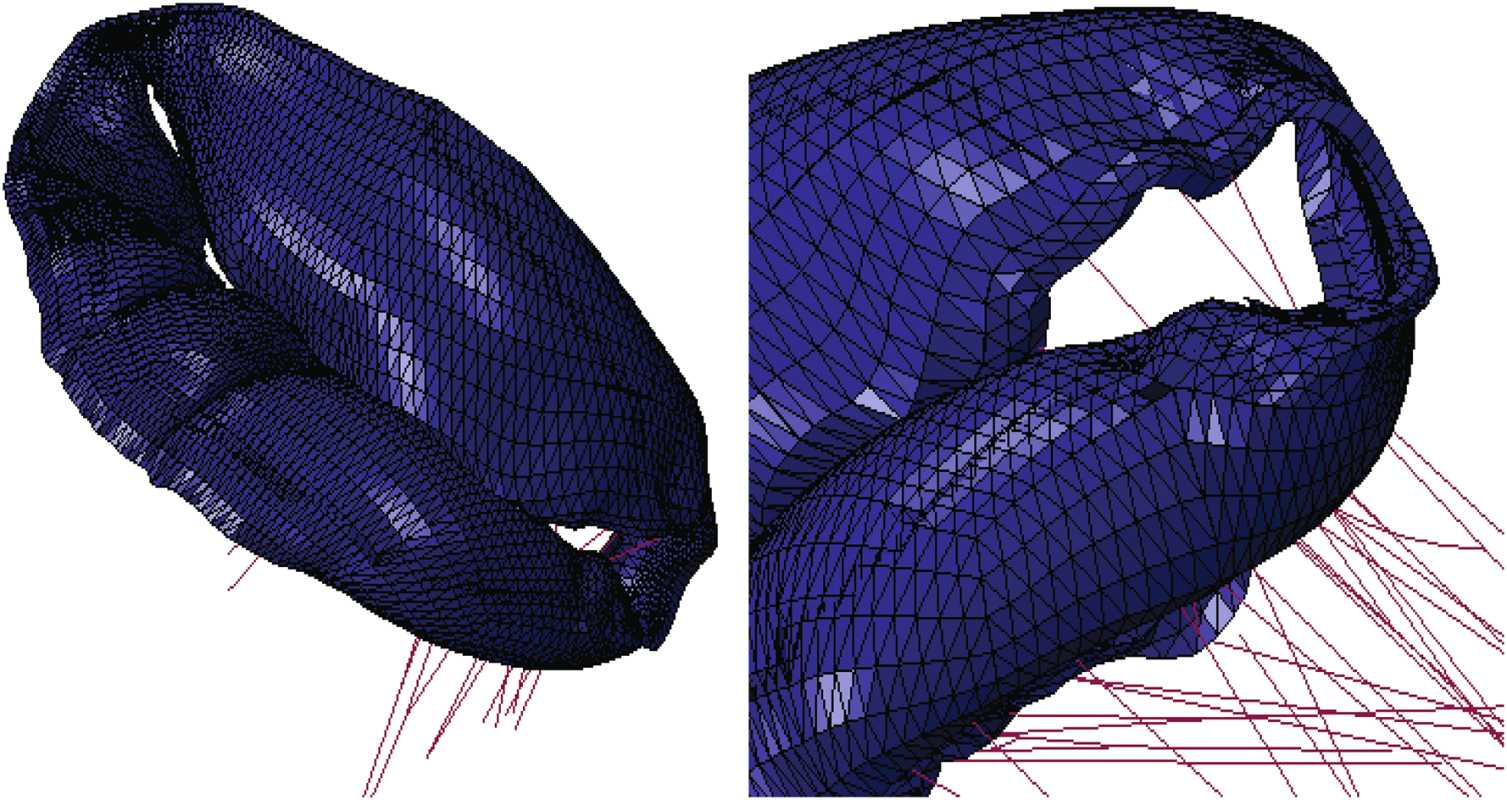
Effect of infarcted LV region on mitral valve leaflet coaptation. Left: view of entire mitral valve; Right: close-up view of affected area

**Figure 15:**
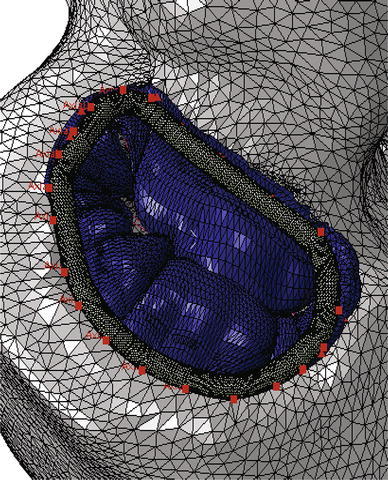
Close-up view of the mitral valve in the whole heart model with a simulated infarcted LV region. The left atrium was removed to clearly show how the simulated novel device increases mitral leaflet coaptation

**Figure 16:**
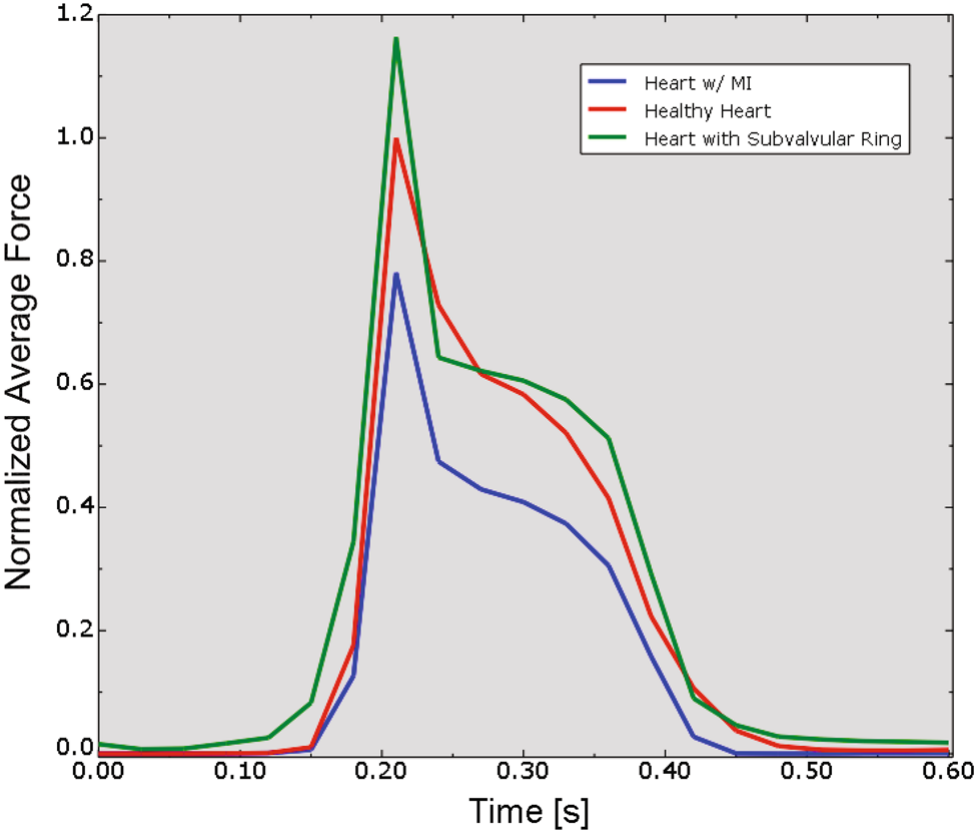
Average forces in chordae between the posterior mitral valve leaflet and the posterior LV papillary muscle during the cardiac cycle. The force time-courses were normalized by the peak average chordae force value in the healthy heart (red curve). Also shown are the cases of LV myocardial infarction (blue curve) and diseased heart treated with our novel device (green curve)

The more realistic geometry of the Generation II Solid Human heart model, and improved diastolic myocardial material properties caused LV ejection fraction to increase from 19.4 to 55%. Figure 11 shows normal LV behavior predicted by this model. Additionally, the model predicted maximum long-axis shortening of 12.1 mm, which is in agreement with the measurements (12.2 ± 3.8 mm) of Rogers *et al*.^16^ Figure 12 shows close-up views of the second generation HCFS with the left atrium removed to clearly show the mitral valve in its open and closed states during the cardiac cycle. Movie 1 shows the same view, but with the mitral valve in action throughout the cardiac cycle. To induce regurgitant mitral valve mechanics, the size of the infarcted LV region was iteratively increased until the LV ejection fraction decreased from 55 to 45%. The effect of myocardial infarction on the LV pressure–volume loop is shown in Fig. 13. Creating the infarcted LV region also resulted in decreased mitral valve leaflet coaptation (Fig. 14). This is shown dynamically in Movie 2. Simulation of the annuloplasty ring with the sub-valvular component resulted in an increase in mitral valve coaptation in the area originally affected by the LV infarction. Figure 15 shows a close-up view of the model with the left atrium removed to clearly illustrate this. Movie 3 shows the same view but with the mitral valve in contact with our novel device throughout the cardiac cycle. The simulation was made with a rigid (flat plane) annuloplasty ring and sub-valvular element, and thus, device stresses and strains cannot be extracted from the analysis. In our opinion, another consequence of using that particular rigid annuloplasty ring is that a non-negligible regurgitant area characterized the post-annuloplasty configuration in the A1-P1 region in Movie 3. On the other hand, we were able to extract average forces (Fig. 16) in the chordae between the posterior mitral valve leaflet and the posterior LV papillary muscle during the cardiac cycle for the three cases (i.e., healthy heart, heart with an LV myocardial infarction, and diseased heart treated with our novel device). After LV myocardial infarction, peak average chordae force became 78% of that in the healthy heart. Treatment of the diseased heart with our novel device caused peak average chordae force to increase, to 116% of that in the healthy heart. Otherwise, average chordae force in the treated case followed that in the healthy case throughout the cardiac cycle.

## Discussion

Our study details three significant advancements in realistic simulation of human cardiac function, pathology, and therapeutic options, for the entire heart. First, incorporating improved cardiac geometry and diastolic myocardial material properties in the Dassault Systèmes human cardiac function simulator (HCFS) caused LV ejection fraction to increase from 19% to a more physiological value of 55%. Second, simulating infarction of the posterior LV papillary muscle caused regurgitant mitral valve mechanics; a pathologic state associated with a substantial risk of death.^2^ Third, use of our undersized annuloplasty ring that included a sub-valvular element corrected valve dysfunction. Figure 10 clearly shows the impact of our novel device; the engagement of the chordae causes the posterior leaflet to move forward, which ultimately causes improved coaptation of the valve leaflets during ventricular systole. Moreover, our results suggest that virtual valve repair, in combination with nonlinear programming tools, e.g., the Dassault Systèmes Abaqus software in conjunction with Isight, can be used to optimize the design of novel devices for correction of ischemic mitral or tricuspid regurgitation. Lastly, the simulations could be repeated with a deformable annuloplasty ring and sub-valvular element, and fatigue-simulation software to make durability predictions.

The most effective surgical approach for treating severe ischemic mitral regurgitation remains controversial.^2^ In the past few years, the use of mitral-valve repair has greatly exceeded the use of replacement.^7^ However, no randomized trials have established the superiority of repair across a spectrum of patients with severe ischemic mitral regurgitation.^2^ In their correspondence, Gorman *et al*.^9^ point out that there was substantial reverse remodeling among the patients in the repair group in Ref. 2 who did not have recurrent moderate or severe mitral regurgitation. They propose that all patients should undergo repair and that those in whom postoperative moderate or severe mitral regurgitation develops should undergo secondary percutaneous mitral-valve replacement. According to Acker *et al*.,^1^ a less speculative and less aggressive approach would be to use predictive models of recurrent mitral regurgitation, and, in patients with a high likelihood of recurrence, to use replacement or a more complex repair technique that specifically addresses leaflet tethering.

Ours is the second study to be performed as part of the Living Heart Project. The first,^3^ which presented a proof-of-concept simulator for a four-chamber human-heart model created from computed tomography and MRI data, illustrated the governing equations of excitation–contraction coupling and discretized them using an explicit finite element environment (Abaqus/explicit).^3^ To illustrate the basic features of their model, the authors visualized the electrical potential and mechanical deformation across the human heart throughout its cardiac cycle. Nine parameters defined the orthotropic passive myocardial response (as measured in excised porcine myocardium by Dokos *et al*.^5^), resulting in an LV ejection fraction of 19.4%. In the present study, we used five different passive mechanical parameter values to define transverse isotropy with respect to the local myofiber direction as observed in the LVs of five normal human subjects, with a resultant ejection fraction of 55%. It might seem quite surprising that these two sets of model parameters (both supposedly representing normal mammalian passive myocardial mechanical properties) predict such different LV ejection fractions. The most likely explanation for this discrepancy is that the excised porcine myocardium studied by Dokos and co-workers was abnormally stiff (in contracture). This explanation seems to indicate that material parameters determined or estimated from *in vivo* clinical or experimental data predict cardiac mechanics more realistically than those directly measured from* ex vivo* experimental data.

Ours is one of the few finite element modeling studies to include the mitral valve and the LV. The first finite element model of the LV with mitral valve^19^ did not include the right ventricle or either atrium. In that study, the authors expanded their previous finite element models of the LV to incorporate the leaflets and chordae of the mitral valve based on drawings of the* ex vivo* ovine mitral apparatus. Their LV model was based on MRI data from a sheep that developed moderate ischemic mitral regurgitation after postero-basal myocardial infarction. They demonstrated the utility and power of their finite element model by using it to test the hypothesis that a reduction in the stiffness of the ischemic region will decrease dyskinesis of the posterior LV wall, increase the displacement of the posterior papillary muscle and thereby increase ischemic mitral regurgitation. However, in that study, the leaflets were not modeled to include contact, which resulted in spurious penetration of the anterior leaflet into the posterior leaflet. That limitation was corrected in a subsequent study of the effect of annuloplasty ring shape in ischemic mitral regurgitation.^12^,^20^ That study concluded that the effects of saddle-shaped and asymmetric mitral annuloplasty rings are similar. This conclusion begs the question as to why are there so many different mitral annuloplasty rings available to cardiac surgeons and more importantly, on what basis have they been designed.^4^,^15^

### Study Limitations and Future Directions

In our study, we made significant improvements in cardiac geometry and diastolic myocardial material properties in the Dassault Systèmes HCFS. Opportunity for added realism in the HCFS remains, however. Our ultimate goal is to replace the compartment approach to the fluids portion of the simulation with a resolved 3D fluid dynamics solution. A fully coupled fluid–structure interaction (FSI) model of the human heart is highly desirable, albeit hugely challenging, and the major focus of research for numerous investigators. Kunzelman and co-workers^6^,^11^,^17^ are utilizing an advanced FSI model of the mitral valve system that allows analysis of the valve in the normal, diseased, or repaired states. Their findings are validated by utilizing a well-established, but unique experimental, *in vitro* system in which mitral valve function can be extensively assessed. Once fully validated, their FSI model can be used to explore and compare various types of valvular pathology and repair. The long-term goal of their research is to provide an advanced FSI model of the mitral valve that could ultimately be used for individualized patient planning for mitral valve repair.

Including flow analysis like that mentioned above would allow us to predict shear stresses on the myocardial wall, and more importantly, on the four heart valves, through the entire cardiac cycle. Such an approach presents tremendous opportunities to better understand the mechanisms of valvular disease and optimize treatment in the form of valve repair or replacement, either through open heart surgery or minimally invasive intervention. Despite the limited treatment of fluids in the current analysis, we are confident that our novel annuloplasty ring with a sub-valvular element will provide durable correction of ischemic mitral regurgitation because of (unpublished) promising long-term outcomes data from treated sheep.

## Conclusion

This study incorporated improved cardiac geometry and diastolic myocardial material properties in the Dassault Systèmes HCFS, which resulted in a realistic LV ejection fraction of 55%. Simulating infarction of the posterior LV papillary muscle predicted regurgitant mitral valve mechanics. Use of our undersized annuloplasty ring that included a sub-valvular element corrected valve dysfunction. Our experience suggests that valve repair can be further optimized with additional software (e.g., fatigue, optimization) to develop novel annuloplasty rings with sub-valvular elements for correction of ischemic mitral or tricuspid regurgitation.

## Electronic supplementary material

Below is the link to the electronic supplementary material.

Supplementary material 1 (MPG 1164 kb)

Supplementary material 2 (MPG 1162 kb)

Supplementary material 3 (MPG 1214 kb)
